# Process evaluation methods and results from the Health in Pregnancy and Postpartum (HIPP) randomized controlled trial

**DOI:** 10.1186/s12884-022-05107-x

**Published:** 2022-10-26

**Authors:** Sara Wilcox, Alicia A. Dahl, Alycia K. Boutté, Jihong Liu, Kelsey Day, Gabrielle Turner-McGrievy, Ellen Wingard

**Affiliations:** 1 Prevention Research Center, Arnold School of Public Health, University of South Carolina, 921 Assembly Street, 29208 Columbia, SC USA; 2 Department of Exercise Science, Arnold School of Public Health, University of South Carolina, 29208 Columbia, SC USA; 3 Department of Public Health Sciences, University of North Carolina at Charlotte, 28105 Charlotte, NC USA; 4 Department of Health Promotion, Education, and Behavior, Arnold School of Public Health, University of South Carolina, 29208 Columbia, SC USA; 5 Department of Epidemiology and Biostatistics, Arnold School of Public Health, University of South Carolina, 29208 Columbia, SC USA

**Keywords:** Pregnancy, Postpartum, Women, Weight, Physical activity, Nutrition, Process evaluation, Implementation, Behavioral intervention

## Abstract

**Background:**

Excessive gestational weight gain has increased over time and is resistant to intervention, especially in women living with overweight or obesity. This study described the process evaluation methods and findings from a behavioral lifestyle intervention for African American and white women living with overweight and obesity that spanned pregnancy (≤ 16 weeks gestation) through 6 months postpartum.

**Methods:**

The Health in Pregnancy and Postpartum (HIPP) study tested a theory-based behavioral intervention (vs. standard care) to help women (N = 219; 44% African American, 29.1 ± 4.8 years) living with overweight or obesity meet weight gain guidelines in pregnancy and lose weight in postpartum. Participants completed process evaluation surveys at 32 weeks gestation (n = 183) and 6 months postpartum (n = 168) regarding their perceptions of most and least helpful aspects of the intervention. A database tracked delivery and receipt of intervention components (in-depth counseling session, telephone calls, podcasts). Descriptive statistics are used to report fidelity, dose, and participants’ perceptions. We also tested whether dose of behavioral intervention components was associated with gestational weight gain and 6-month postpartum weight retention with linear regression models controlling for baseline age and gestational weeks, receipt of Medicaid, race, parity, and marital status. A content analysis was used to code and analyze responses to open-ended survey questions.

**Results:**

Over 90% of participants (both groups) would recommend the program to a friend. Implementation fidelity was moderately high and greater in pregnancy than postpartum for all intervention components. Dose received and participants’ ratings of the in-depth counseling session and telephone calls were more favorable than podcasts. The Facebook group was not perceived to be very helpful, likely because of low participant interaction. Although podcasts were created to reinforce call topics, this redundancy was viewed negatively by some. More calls completed and more podcasts downloaded related to lower gestational weight gain (p < .05).

**Conclusion:**

Study findings underscore challenges in engaging this important but busy population, especially during the postpartum period.

**Trial registration:**

The study was registered at clinicaltrials.gov (NCT02260518) on 10/09/2014. https://clinicaltrials.gov/ct2/show/NCT02260518.

## Background

Women who enter pregnancy living with overweight or obesity are at increased risk for deleterious health outcomes [1], and promoting healthy lifestyle behaviors including regular physical activity, healthy nutrition, and healthy weight gain in all women, regardless of their weight status, is recommended [1–5]. Pregnant women are less active than non-pregnant women, and physical activity is consistently shown to decline over the course of pregnancy [6]. Although studies of natural changes in dietary habits during pregnancy are lacking [7], nearly half of women exceed the recommended weight gain guidelines during pregnancy, and excessive gestational weight gain is more common among women already living with overweight and obesity at the start of their pregnancy [8].

Evidence is accumulating that lifestyle interventions (physical activity, diet, weight gain counseling) not only reduce excessive gestational weight gain, but also result in more favorable birth outcomes. For example, a recent systematic review found that physical activity and dietary interventions in general samples of pregnant women were associated with less gestational weight gain relative to standard care (-1.15 kg), with evidence stronger for diet alone (-2.63 kg) than physical activity alone (-1.04 kg) interventions [9]. Furthermore, this review demonstrated a reduced risk of adverse maternal and neonatal outcomes such as gestational diabetes and cesarean section. Similarly, another systematic review found that counseling and behavioral interventions for healthy weight and weight gain during pregnancy yield modest reductions in gestational weight gain and are associated with reduced risk of several maternal and neonatal outcomes [10].

Fewer interventions have focused on women living with overweight and obesity. A 2019 review found that physical activity interventions during pregnancy for women living with overweight or obesity yielded slight improvements in physical activity outcomes, but methodological limitations including inadequate reporting of intervention dose limited conclusions [11]. Another review of 21 randomized trials of dietary and/or physical activity interventions among women who were living with overweight or obesity found that dietary interventions resulted in less gestational weight gain than physical activity only or combined interventions [12]. It is noteworthy that 19 of the 21 studies were evaluated as being high in methodological risk. More recently, Cantor et al.’s review [10] showed that improvements in birth outcomes were seen across weight status categories, but interventions yielded more favorable outcomes in gestational weight gain for women living with obesity relative to women in other weight classifications.

Behavioral interventions during pregnancy often do not report process evaluation outcomes such as fidelity and dose [11]. These types of process outcomes are critical for understanding what components of interventions are delivered and received, what components are most and least preferred, and how these process outcomes might relate to study outcomes. To address this gap in the literature, we conducted a process evaluation of the Health In Pregnancy and Postpartum (HIPP) study, a randomized clinical trial evaluating the impact of a lifestyle intervention (versus standard care) on gestational weight gain and related outcomes in African American and white women who enter pregnancy living with overweight or with obesity. This study is unique in that it intervened from pregnancy (≤ 16 weeks gestation) through 6 months postpartum and recruited a sample of both African American and white women living with overweight or obesity.

## Methods

### Aims

The aim of the Health In Pregnancy and Postpartum (HIPP) study, a randomized clinical trial, was to evaluate the impact of a lifestyle intervention (versus standard care) on gestational weight gain (primary outcome) and related outcomes in African American and white women who enter pregnancy living with overweight or obesity [13]. In earlier papers, we described the study design and methods, including the intervention [13], as well as recruitment strategies and yields [14] and the impact of the intervention on gestational weight gain [15] and postpartum weight retention [16]. In our two outcome papers [15, 16], we reported associations between receipt of counseling calls and gestational weight gain / postpartum weight retention. However, these papers did not report other components of intervention delivery overall or with respect to weight-related outcomes. Furthermore, they did not report participants’ experiences with the intervention nor factors associated with receipt of intervention components. Finally, we have not reported delivery of the standard care program components nor participants’ experiences of them. Therefore, the purpose of the current paper is to report the methods used in the process evaluation of the trial and the process evaluation findings with respect to receipt of intervention components (i.e., fidelity) for both the intervention and standard care groups. We also report participants’ perceptions of most and least helpful aspects of these components. Finally, we present sociodemographic variables associated with receipt of the intervention components and the associations between receipt of all of the intervention components (i.e., dose) and weight-related outcomes during pregnancy and postpartum.

## Design, setting, and participants

The Institutional Review Boards from three participating healthcare centers and one university approved the study protocol. All participants provided written informed consent before participating. All methods were carried out in accordance with the Declaration of Helsinki and with the Institutional Review Boards that approved the study. The study was registered at clinicaltrials.gov (NCT02260518) on 10/09/2014. A detailed description of the HIPP study design and methods is available elsewhere [13]. Participants (N = 219), recruited largely via obstetric and gynecology clinics in South Carolina [14], were randomized to either a behavioral lifestyle intervention (n = 112) or a standard care condition (n = 107). Measurement staff were blind to randomization assignment. Participants completed assessment visits at baseline (≤ 18 weeks gestation), 32 weeks gestation, and 6 months postpartum. A modest monetary incentive was provided for each visit completed. Women were eligible if they were ≤ 16 weeks of a singleton pregnancy, had a body mass index (BMI) ≥ 25.0 kg/m^2^, were 18–44 years of age, were white or Black / African American, were able to read and speak English, had no plans to move outside geographic area in the next 18 months, agreed to be randomized, did not have health conditions that made exercise or dietary change unsafe, were not hospitalized for a mental health or substance-abuse disorder in the past 6 months, and were able and willing to take part in the intervention activities (e.g., regular access to a telephone). Characteristics of study participants are shown in Table 1. Close to half of participants were African American (44%), 59% were college graduates, and their average age was almost 30 years. Just over half of participants (52%) were categorized as living with obesity based on their pre-pregnancy BMI, with the remainder living with overweight. Participants, on average, enrolled in the study at 13 weeks gestation, and for 43% of participants, this was their first pregnancy.

**Table 1:** Baseline characteristics of study participants (N = 219), overall and by randomization group

	All participants (N = 219)	Behavioral intervention (n = 112)	Standard care (n = 107)
	% or Mean (SD)	% or Mean (SD)	% or Mean (SD)
Race			
White	55.7	58.0	53.3
African American	44.3	42.0	46.7
Age, years	29.7 (5.0)	30.4 (5.2)	29.1 (4.8)
18–24	15.9	12.5	19.6
25–29	28.3	27.7	29.0
30–34	37.4	39.3	35.5
≥ 35	18.3	20.5	15.9
Education			
Grades 9–11	1.4	0	2.8
Grade 12 or GED	11.0	8.0	14.0
College 1–3 years	28.3	32.1	24.3
College 4 years or more	59.4	59.8	58.9
Family Income			
< $35,000	29.4	23.4	35.5
35,000 - $49,999	13.8	15.3	12.1
50,000 - $74,999	19.3	18.0	20.6
≥ $75,000	37.6	43.2	31.8
Employment during pregnancy			
Employed full-time	61.2	61.6	60.8
Part-time or unemployed	38.8	38.4	39.2
Marital status*			
Married	67.1	75.0	58.9
Unmarried	32.9	25.0	41.1
Pre-pregnancy body mass index, kg/m^2^	33.4 (6.4)	33.0 (6.6)	33.9 (6.1)
Overweight	48.4	50.0	46.7
Obese	51.6	50.0	53.3
Gestational age, weeks	12.6 (2.3)	12.7 (2.4)	12.5 (2.2)
Parity			
0	42.9	43.8	42.1
1	36.5	34.8	38.3
≥ 2	20.6	21.4	19.6

^*^Unmarried included divorced (n = 8), separated (n = 7), and never married (n = 57) women

## Process evaluation methods and measures

The process evaluation was designed to: (a) examine the extent to which the intervention took place and in a timely manner, (b) examine whether intervention activities were consistent with the underlying behavioral theory, (c) provide guidance concerning necessary adjustments to the intervention, and (d) monitor intervention fidelity.

### Dose received

Intervention staff recorded all activities in a Microsoft Access database developed for the study. Staff recorded the date, start and end time, location, and general notes regarding the in-depth counseling sessions and telephone calls. Further, they recorded the dates that podcast emails and mailings were sent and the dates that participants downloaded the podcasts. This information allowed us to determine the dose received for primary intervention components.

### Participant ratings

Behavioral intervention participants and standard care participants completed a process evaluation survey at the 32-week gestation and the 6-month postpartum measurement visit. Unlike other study measures that were interviewer-administered, the process evaluation surveys were provided in an envelope and self-administered and returned in the same envelope to try to reduce social desirability bias. These comprehensive surveys assessed various domains related to participants’ experiences with HIPP. Participants’ satisfaction with HIPP was assessed with questions regarding their overall rating of the program, their rating of their interventionist (behavioral intervention only), whether they would recommend the program to a friend, and a self-rating of their effort (behavioral intervention only). The perceived helpfulness of each intervention component was assessed with Likert-style ratings. Participants in the behavioral intervention group also reported how frequently they used each of the program’s behavioral strategies and how helpful they found each strategy.

### Open-ended questions

The pregnancy and postpartum process evaluation surveys asked participants to answer open-ended questions about what they liked the most and least (or found most or least helpful) about each of the intervention components and whether they wished to share anything else about the program. The postpartum process evaluation survey also asked participants what would have helped them take part in the program more fully. Only responses from the behavioral intervention participants are presented in this paper.

## Description of behavioral lifestyle intervention group

*Behavioral goals and theory*. The behavioral lifestyle intervention was designed to support participants in meeting recommendations for physical activity, dietary intake, healthy weight gain during pregnancy, and healthy weight loss during postpartum [4, 17–19]. These recommendations were to accumulate 150 min per week of moderate intensity physical activity; eat a diet high in fruits, vegetables, and whole grains and low in saturated fats, while also balancing caloric intake to match but not exceed dietary needs for pregnancy and lactation; gain weight consistent with the 2009 Institute of Medicine guidelines during pregnancy [5]; and lose 1–3 pounds per week during postpartum. The intervention was grounded in Social Cognitive Theory [20] and was tailored to the unique needs, interests, and barriers of pregnant women, as guided by the literature [21, 22] and our own formative work [23, 24]. Specific evidence-based strategies included behavioral skills and knowledge, self-efficacy (via setting realistic and progressive goals), self-regulation (self-monitoring, goal setting, problem solving, use of reinforcement), stimulus control (prompting of healthier choices), seeking out social support, time management, and relapse prevention.

*In-depth counseling sessions*. The intervention began with an in-depth counseling session (~ 60 min) where the study interventionist provided a detailed, printed feedback report regarding the participant’s baseline dietary intake (based on two 24-hour dietary recalls) and physical activity (based on SenseWear armband data from at least 5 days of wearing). Most of these sessions were conducted in person at the university, but telephone and home options were provided as needed. Current behaviors relative to study goals and national recommendations were highlighted. The MyPlate Daily Checklist for Moms was used to support nutrition content, based on pre-pregnancy BMI and tailored to each trimester. Each participant received a personalized weight gain tracking chart that displayed upper and lower recommended weight gain lines drawn over time (i.e., gestational weeks). Participants were encouraged to weigh themselves (personal scale provided to each participant) and track their weight gain on the chart weekly. Initial dietary and physical activity goals were established. Participants received a binder of study handouts corresponding to the educational content covered on the first 10 telephone counseling calls. They also received a pedometer to track physical activity (i.e., daily steps).

At 6 to 8 weeks postpartum, a second in-depth counseling session was provided (~ 60 min). The postpartum session focused on setting goals for resuming physical activity (after being cleared by their health care provider), meeting nutritional needs during postpartum (including for those who were breastfeeding), setting a weight loss goal, and discussing strategies for losing 1 to 3 pounds per week. MyPlate Daily Checklist for Moms guided the postpartum nutrition content, which was tailored to breastfeeding status. All participants received a personalized weight loss tracking graph with the upper (3 pounds per week) and lower (1 pound per week) bounds of recommended weight loss over time for six months (adjusted to not go below a healthy BMI).

*Telephone counseling*. Based on what we learned about participant preferences during our formative work [23, 24], the intervention began with 10 weekly in-person group sessions. However, due to recruitment challenges and poor attendance, the protocol was changed to convert these 10 group sessions to 10 individual telephone counseling calls (content remained the same). This change allowed for rolling recruitment of participants. Only 6 participants took part in the group sessions before the protocol change. Each of the first 10 calls during pregnancy was designed to last about 20 min and began with an assessment of whether there had been any health-related or pregnancy-related changes. Participants were asked to report their weight and plot it on their weight gain graph (the interventionist did the same) which led to a discussion of how their weight gain compared to the IOM recommendations. Each call emphasized at least one key behavioral strategy (described earlier) applied to physical activity and healthy eating and included didactic information and discussion, for example, pregnancy myths (e.g., you need to eat for two, physical activity is unsafe), safety considerations for physical activity, and dietary education (e.g., how to know which grains are whole grains). Participants were often referred to their binder and handouts during these calls. Calls ended with setting a weekly physical activity and healthy eating goal. Participants who completed the call and downloaded the corresponding podcast (described later) received a small incentive (e.g., baby products) for that week.

After the first 10 weekly pregnancy calls ended, participants received shorter (designed to be 10 to 15 min) weekly or every other week (depending on their preference) counseling calls until delivery. These calls assessed safety considerations, collected and plotted current weight, discussed progress toward physical activity and healthy eating goals set during the previous call, problem-solved barriers to reaching goals (as needed), and set goals for the upcoming week(s).

After participants delivered, they received very brief weekly check-in calls until the in-depth counseling session was delivered. These supportive calls were intended to last only a couple of minutes, and included no discussion of weight, physical activity, or diet. After the in-depth postpartum counseling session at 6–8 weeks, and through 6 months postpartum, participants were contacted for biweekly telephone counseling calls. Although these calls focused on weight loss rather than healthy weight gain, the structure and format of the calls were the same as the brief pregnancy calls described earlier.

*Behavioral podcasts*. To reinforce behavioral strategies and content covered on telephone calls, and to present this information in a convenient (i.e., can be accessed at any time) and engaging format, participants received 10 podcasts during pregnancy and 16 during postpartum. Just prior to each of the first 10 pregnancy calls, staff emailed or send a text message to participants with a link to the podcast for the week and the handouts that corresponded with the call (duplication of binder materials). These messages were scheduled with Boomerang for Gmail. Each podcast paralleled the content of the corresponding phone call and provided information on recommended weight gain in pregnancy, healthy eating, and physical activity. Podcasts averaged 21 min in duration and featured scripted character narratives for two pregnant women attempting healthy lifestyle changes and healthy weight gain. The main characters were portrayed by two voice actors; one African American and one white woman. The podcast recordings also included a narrator to guide the storyline and connect module content. The voice actors reviewed the previous week’s topic and provided information consistent with the topics covered in that call (i.e., healthy weight gain, physical activity, nutrition, behavioral strategy). The podcasts were based on previous work that demonstrated their efficacy for weight loss in non-pregnant adults [25].

Beginning at 4 weeks postpartum, participants received a link to their first of 16 weekly podcasts that followed the 16 core Diabetes Prevention Program sessions and focused on gradual weight loss (1–3 pounds per week) [26]. Where necessary, the content was tailored for postpartum women. Like the pregnancy podcasts, the main character storylines were portrayed by two voice actors, one African American and one white woman, and included a narrator. Podcasts averaged 20 min in duration.

Participants were required to enter their study ID and initials to download each podcast. This procedure allowed the study to track whether the participant downloaded that week’s podcast. Reminder emails were sent to participants who had not downloaded the podcast. If participants did not have a device that allowed them to access the podcast, the study sent a CD with that week’s podcast on it (1 participant was excluded from analyses that reported the percentage who downloaded postpartum podcasts).

*Private Facebook group*. Participants were encouraged to join a private Facebook group for pregnancy during the in-depth pregnancy counseling session and a private Facebook group for postpartum during the in-depth postpartum counseling session. Both groups were moderated by intervention staff. Participants remained in the Facebook pregnancy group until 6 to 8 weeks postpartum and then transitioned to the postpartum group. The Facebook postpartum group continued through 6 months postpartum. Intervention staff posted one message per day (Monday through Friday) that reinforced behavioral skills, healthy weight gain in pregnancy, modest weight loss in postpartum, physical activity (including exercise videos for pregnancy and postpartum), and/or diet (including links to recipes). The content for both Facebook groups was posted on a continuous cycle, but with enough content to prevent anything from being repeated for a given participant (i.e., 40 weeks x 5 posts/week = 200 posts for pregnancy, and 24 weeks x 5 posts/week = 120 posts for postpartum). Content was not matched to gestational age or weeks postpartum as enrollment into HIPP was rolling. Posts were prescheduled using Hootsuite. Participants could respond to posts from study staff as well as post content to the group directly as a group member (i.e., peer-to-peer posting was enabled), with monitoring of posts by staff.

## Description of standard care group

Participants assigned to the standard care group attended regularly scheduled clinic visits with their prenatal care provider. To enhance retention and participant engagement, standard care participants also received study mailings of publicly available educational materials and podcasts. During pregnancy, the six study mailings (one per month) focused on tips for a healthy pregnancy as well as fetal development. During postpartum, the six study mailings (one per month) focused on infant development. Standard care participants also received 10 weekly podcasts during pregnancy and 16 weekly podcasts during postpartum. Their timing corresponded to the delivery of the behavioral lifestyle intervention podcasts. Pregnancy podcasts averaged 28 min, and postpartum podcasts averaged 22 min. Podcasts focused on having a healthy pregnancy, fetal and infant development, and parenting but avoided content related to nutrition, physical activity, pregnancy weight gain, and postpartum weight loss. A small incentive was provided to participants who listened to at least 9 of the 10 pregnancy podcasts. Podcast emails and tracking were identical to what was described earlier for the behavioral lifestyle intervention (7 participants were excluded from analyses that reported the percentage who downloaded podcasts).

## Outcome measures

Total gestational weight gain in kilograms was the primary outcome for the HIPP trial [15]. It was calculated as the difference between the medically abstracted weight recorded at delivery and self-reported pre-pregnancy weight reported on the initial screening questionnaire (available for all 112 behavioral intervention participants). When delivery room weight was not available, weight at the last prenatal care visit was used (n = 56), where the mean gestational age was 38.5 weeks, an average of 4.8 days earlier than gestational age at delivery.

Weight retention in kilograms at 6 months postpartum was calculated as the difference between weight assessed at the 6-month assessment visit (conducted by HIPP study staff) and self-reported pre-pregnancy weight reported on the initial screening questionnaire (available for 83 of 112 behavioral intervention participants).

## Statistical analyses

Descriptive statistics (means, standard deviations, percentages) were used to summarize the key indicators in the process evaluation. We tested, separately by randomization group, whether those who completed the 32-week pregnancy and the 6-month postpartum process evaluation survey differed from those who did not by race, education, income, employment, marital status, weight, and parity using Fisher’s Exact Tests and by age and BMI using t-tests. We also examined whether survey completion rates were associated with each intervention component (in-depth counseling sessions, call completion, podcasts downloaded, and Facebook group).

We studied whether receipt of the intervention components (in-depth counseling session, telephone calls, podcasts, and Facebook group) for behavioral intervention participants differed by these same dichotomous and continuous sociodemographic variables described in the previous paragraph.

We tested whether the dose of behavioral intervention components during pregnancy was associated with the primary study outcome of gestational weight gain. We first examined correlations between each intervention component and gestational weight gain. We then conducted linear regression models that controlled for the same covariates used in our primary outcomes paper [15]: age at baseline, gestational weeks at baseline, Medicaid recipient (yes/no), race, parity, and marital status, as defined earlier. These analyses were repeated to examine associations between receipt of the behavioral intervention components during postpartum and 6-month postpartum weight retention.

For the open-ended questions, participants’ written responses were typed verbatim. Responses tended to be brief and lacked some of the richness typically seen in focus groups or in-depth interviews. Thus, we exported responses to Microsoft Excel and used content analysis to code responses question-by-question [27, 28], using columns to record the assigned code(s). Open codes were created, and codes were assigned to each response (as appropriate, multiple codes were used for a single response). Over the course of the study, two different people, AB and KD, did initial coding. One of the study PIs (SW) reviewed all codes, provided feedback, and discussed any differences in opinions. The questions were then recoded and reviewed a second time by SW, and differences were again discussed, and consensus reached. Responses for each code were then ordered according to the frequency with which each code was used. To identify the most prevalent themes, we chose to focus on themes that emerged from at least 10% of respondents (9 or more participants on the pregnancy survey, 8 or more participants on the postpartum survey). These criteria appeared to distinguish natural breaks in the frequency of codes.

## Results

### Completion of process evaluation surveys

The 32-week pregnancy process evaluation survey was completed by 88 (78.6%) behavioral intervention and 96 (89.7%) standard care participant participants. An additional 4 behavioral intervention and 2 standard care participants completed the 32-week visit but did not complete the process evaluation survey. Among behavioral intervention participants, survey completion did not differ by race, education, income, employment, marital status, weight status, parity, age, or BMI. Those who completed the 32-week pregnancy process evaluation survey were more likely to have completed the in-depth pregnancy counseling session than those who did not (83.8% vs. 0%, p < .0001), received more of the content-based telephone counseling calls during pregnancy (9.36 vs. 2.58, t = -8.72, p < .001), and downloaded more pregnancy podcasts (8.50 vs. 2.46, t = -8.00, p < .0001). Among the standard care participants, pregnancy survey completion rates were higher among college graduates versus those with less than a college degree (96.8% vs. 81.8%, p < .05), participants employed full time versus not employed full time (95.4% vs. 83.3%, p < .05), and those who were married versus not married (96.8% vs. 81.8%, p < .05). Those who completed this survey also had a lower BMI than those who did not (32.40 vs. 36.60, t = 2.11, p < .05). There were no differences by race, income, weight status, parity, or age.

The 6-month postpartum process evaluation survey was completed by 78 (69.6%) behavioral intervention and 90 (84.1%) standard care participants. An additional 5 behavioral intervention and 1 standard care participants completed the 6-month visit but did not complete the process evaluation survey. Among behavioral intervention participants, 6-month postpartum survey completion rates were higher among college graduates versus those with less than a college degree (77.6% vs. 57.8%, p < .05) and among married versus unmarried participants (76.2% vs. 50.0%, p < .05). There were no differences by race, income, employment, weight status, parity, age, or BMI. Those who completed the 6-month postpartum process evaluation survey also were more likely to have completed the in-depth postpartum counseling session than those who did not (88.5% vs. 4.0%, p < .0001), received more postpartum telephone counseling calls (11.35 vs. 1.82, t = -14.85, p < .001), and downloaded more postpartum podcasts (7.22 vs. 0.52, t = -8.77, p < .0001). Among standard care participants, college graduates were significantly more likely than those with less than a college degree to complete the postpartum process evaluation survey (92.1% vs. 72.7%, p < .05), but completion rates did not differ by race, income, employment, marital status, weight status, parity, age, or BMI.

## Overall ratings of the program

Overall, as shown in Table 2, both intervention and standard care participants rated the HIPP program favorably in pregnancy and postpartum, with a mean rating of 3.4 out of 4, indicating a rating between “good” and “excellent.” Similarly, over 90% of participants in both groups at both time periods said they would recommend the program to a friend. Intervention participants rated their health educator very favorably (3.7 out of 4), nearly as “excellent,” but rated their own effort and ability to meet goals as markedly lower (2.7 and 2.6 out of 4), indicating a rating between “good” and “very good” (not assessed in the standard care group).

**Table 2:** Overall ratings of the program, by intervention group

	Behavioral Intervention (n = 88 pregnancy, n = 78 postpartum)	Standard Care (n = 96 pregnancy, n = 90 postpartum)
Question	M (SD) or %	M (SD) or %
Overall rating of the HIPP program (^a^Range: 1–4)﻿		
Pregnancy	3.4 (0.6)	3.4 (0.6)
Postpartum	3.4 (0.7)	3.5 (0.6)
Would recommend program to friend, %		
﻿Pregnancy	94.3	90.6
Postpartum	92.3	96.6
Rating of health educator, pregnancy (^b^Range: 0–4)	3.7 (0.7)	---
Self-rating of effort put into making changes		
Pregnancy (^a^Range: 0–4)	2.7 (0.8)	---
Postpartum (^b^Range: 1–4)	2.6 (0.7)	---
Self-rating of ability to meet goals set with health educator since having baby (^a^Range: 1–4)	2.5 (0.8)	---

^a^Possible Range: 1 = poor, 2 = ok, 3 = good, 4 = excellent

^b^Possible Range: 0 = poor, 1 = fair, 2 = good, 3 = very good, 4 = excellent

## Intervention dose

The program dose received, for each program component, is reported in Table 3. Sociodemographic differences in receipt of the behavioral intervention components are shown in Table 4.

**Table 3:** Program dose received, by intervention group

	Behavioral Intervention (n = 112)	Standard Care (n = 107)
Program Component	M (SD) or %	M (SD) or %
**In-depth counseling session**		
Received session, %		
﻿Pregnancy	93.8	---
Postpartum	77.7	---
Duration of session, minutes		
﻿Pregnancy (n = 105)	60.0 (10.9)	---
Postpartum (n = 87)	46.7 (13.0)	---
**Telephone calls**		
Pregnancy calls delivered – calls 1 to 10	7.9 (3.7)	---
0 calls	9.8	---
1 to 4 calls	11.6	---
5 to 8 calls	4.5	---
9 to 10 calls	74.1	---
^a^Pregnancy calls delivered – calls 11+	4.6 (3.8)	
Postpartum calls delivered (14 to 16 possible)	8.4 (5.4)	---
0 calls	19.6	---
1 to 4 calls	6.2	---
5 to 8 calls	17.0	---
9 to 12 calls	23.2	---
13 to 16 calls	33.9	---
Average duration of calls, minutes		
^ b^Pregnancy calls 1 to 10 (n = 97﻿)	25.4 (6.3)	---
Pregnancy calls 11+ (n = 86)	12.4 (4.8)	---
Postpartum calls (n = 90)	10.4 (3.4)	---
**Podcasts**		
Podcasts downloaded – pregnancy (10 possible)	7.2 (3.7)	^c^6.4 (4.2)
0 podcasts	10.7	18.0
1 to 4 podcasts	14.3	17.0
5 to 8 podcasts	15.2	11.0
9 to 10 podcasts	59.8	54.0
Podcasts downloaded – postpartum (16 possible)	^d^5.2 (6.2)	^c^6.2 (6.8)
0 podcasts	41.4	38.0
1 to 4 podcasts	17.1	18.0
5 to 8 podcasts	10.8	8.0
9 to 12 podcasts	10.8	4.0
13 to 16 podcasts	19.8	32.0
**Facebook group (self-reported)**		
Visited Pregnancy Facebook group (n = 88﻿)	68.2	---
Viewed/posted to Postpartum Facebook group (n = 78﻿)	45.4	---

^a^The number of possible pregnancy calls varies by participant depending on when she entered study and whether she elected to have weekly or bi-weekly calls after the first 10 calls

^b^Six participants who started the intervention with group sessions were not included in call duration analyses for pregnancy call 1 to 10

^c^Seven participants were deleted from analysis because they requested that podcasts be mailed on a CD, so we were unable to track downloads

^d^One participant was deleted from analysis because she requested that podcasts be mailed on a CD, so we were unable to track downloads

**Table 4:** Sociodemographic characteristics associated with receipt of the behavioral intervention components (N = 112)

	Completed In-depth Counseling Sessions		Received 9 or 10 Counseling Calls		Downloaded Podcasts (9–10 Pregnancy; 1 + Postpartum)		Facebook Group	
	% Yes Pregnancy	% Yes Postpartum	% Yes Pregnancy	% Yes Postpartum	% Yes Pregnancy	% Yes Postpartum	^a^ % Yes Pregnancy	^b^ % Yes Postpartum
All participants (N = 112)	93.8	77.7	74.1	57.1	59.8	58.6	68.2	45.4
Race								
White	95.4	80.0	76.9	58.5	63.1	59.4	75.5	51.0
African American	91.5	74.5	70.2	55.3	55.3	57.4	57.1	35.7
Education								
College graduate	92.5	80.6	77.6	62.7	62.7	71.6**	71.2	51.0
Not college graduate	95.6	73.3	68.9	48.9	55.6	38.6	63.9	34.6
Income								
≥ $50,000	94.1	80.9	76.5	63.2	67.6	66.2*	76.4*	49.0
< $50,000	93.0	72.1	69.8	46.5	48.8	45.2	53.1	36.0
Employment								
Employed full time	94.2	82.6	82.6**	62.3	66.7	66.2*	58.9*	42.3
Not employed full time	93.0	69.8	60.5	48.8	48.8	46.5	84.4	52.0
Marital status								
Married	94.1	81.0	78.6	63.1*	65.5*	62.6	76.1**	49.2
Not married	92.9	67.9	60.7	39.3	42.9	46.4	42.9	28.6
Weight status								
Overwei﻿ght	96.4	76.8	71.4	55.4	66.1	56.4	69.6	42.5
Ob﻿ese	91.2	78.6	76.8	58.9	53.6	60.7	66.7	48.6
Parity								
﻿0	91.8	73.5	69.4	55.1	55.1	52.1	59.5	43.7
≥ ﻿1	95.2	81.0	77.8	58.7	63.5	63.5	74.5	46.7

^a^N is limited to those who completed the 32-week gestation process evaluation survey (n = 88)

^b^N is limited to those who completed the 6-month postpartum process evaluation survey (n = 77)

*p < .05 for group difference. **p < .01 for group difference

### In-depth counseling sessions

Almost all behavioral intervention participants (94%) received the in-depth pregnancy counseling session, which lasted an average of 60 min (range: 40 to 106) and occurred, on average, at 16.6 ± 2.5 weeks gestation. Most sessions were conducted at the university (85%), with the remaining conducted via telephone (8%), at the participants’ homes (6%), or at some other location (2%) (data not shown). A smaller percentage (78%) received the in-depth postpartum counseling session, which lasted an average of 47 min (range: 21 to 110). These sessions were conducted at the university (48%), the participants’ homes (37%), and via telephone (15%) (data not shown). There were no significant differences by race, education, income, employment, marital status, weight status, or parity for those who received either the pregnancy or postpartum in-depth counseling sessions versus those who did not (see Table 4). There were also no differences by age or BMI (data not shown).

### Telephone counseling

Behavioral intervention participants completed an average of 8 of the 10 content-based calls, and the calls averaged 25 min (range: 11 to 42). Nearly three-quarters of participants completed 9 or 10 of the calls, although 10% completed no calls. Call 1 occurred, on average, at 17.8 ± 2.6 weeks gestation, and call 10 occurred, on average, at 27.3 ± 3.0 weeks gestation. Participants completed an average of 5 of the shorter pregnancy counseling calls (calls 11+), and they averaged 12 min (range: 4 to 32). Because participants could choose between weekly or biweekly follow-up calls until they delivered and because participants entered the study and delivered their babies at different gestational ages, the total number of possible calls differed by person. Those who were employed full-time were more likely than those not employed full-time to receive 9 or 10 of the 10 content-based calls, but there were no significant differences by race, education, income, marital status, weight status, or parity (see Table 4). Participants who received 9 or 10 calls were significantly older than those who received less than 9 calls (31.0 vs. 28.4 years, p < .05), but there were no differences by BMI (data not shown).

During postpartum, participants completed an average of 8 of the 14 to 16 possible calls. Approximately one-third completed 13 to 16 calls, while 20% completed none of the postpartum calls. By design, these calls were shorter than the calls in pregnancy, lasting an average of 10 min (range: 4 to 20). Married participants were significantly more likely than unmarried participants to receive at least 9 postpartum calls, but there were no significant differences by race, education, income, employment, weight status, or parity (see Table 4). There were also no differences by age or BMI (data not shown).

### Podcasts

Behavioral intervention participants downloaded an average of 7 and standard care participants 6 of 10 possible pregnancy podcasts. While a similar percentage in each group downloaded 9 to 10 podcasts (60% behavioral intervention, 54% standard care), a larger percentage of standard care participants downloaded no podcasts (18% vs. 11%). Among behavioral intervention participants, married participants were more likely to have downloaded 9 or 10 of the pregnancy podcasts as compared to unmarried participants, but there were no significant differences by race, education, income, employment, weight status, or parity (see Table 4). Behavioral intervention participants who downloaded 9 or 10 podcasts were also significantly older than those who downloaded less than 9 podcasts (31.3 vs. 29.0 years, p < .05), but there were no differences by BMI (data not shown).

Substantially fewer postpartum podcasts were downloaded: 5 and 6 of 16 possible podcasts for behavioral intervention and standard care participants, respectively. A large percentage of participants in both groups downloaded none of the postpartum podcasts (41% behavioral intervention, 38% standard care). Fewer behavioral intervention participants (20%) downloaded 13 to 16 of the podcasts relative to standard care participants (32%). Among behavioral intervention participants, college graduates (vs. not college graduates), those with incomes ≥ $50,000 (vs. < $50,000), and those employed full-time (vs. not employed full time) were more likely to have downloaded at least one postpartum podcast; there were no significant differences by race, marital status, weight status, or parity (see Table 4). Participants who downloaded at least one postpartum podcast were significantly older than those who downloaded no postpartum podcasts (31.2 vs. 29.2 years, p < .05), but there were no differences by BMI (data not shown).

### Facebook

We did not formally track participation in the pregnancy and postpartum Facebook groups; however, 68% reported visiting the pregnancy Facebook group and 45% reported viewing or posting to the postpartum Facebook group. Those with incomes ≥ $50,000 (vs. < $50,000), not employed full-time (vs. employed full time), and married (vs. unmarried) were more likely to have visited the pregnancy Facebook group, but there were no differences by race, education, weight status, or parity (see Table 4). There were no differences by race, education, income, employment, marital status, weight status, or parity in viewing or posting to the postpartum Facebook group (see Table 4). There were also no differences by age or BMI (data not shown). No study Facebook group was offered to standard care participants.

## Associations between intervention dose and weight-related outcomes

Gestational weight gain was significantly and negatively correlated with completion of the pregnancy in-depth counseling session (r = -.35, p = .0002), number of pregnancy content calls completed (r = -.23, p = .01), and number of pregnancy podcasts downloaded (r = -.22, p = .02), but was not associated with number of pregnancy calls after the first 10 content calls (r = -.10, p = .27) or visiting the pregnancy Facebook group (r = .03, p = .76). After controlling for covariates, the significant effects remained statistically significant for the completion of the pregnancy in-depth counseling session (parameter estimate = -9.80, t = -3.76, 95% CI = -14.97 to -4.64, p = .0003), number of pregnancy content calls completed (parameter estimate = -0.42, t = -2.23, 95% CI = -0.79 to -0.04, p = .028), and number of pregnancy podcasts downloaded (parameter estimate = -0.43, t = -2.27, 95% CI = -0.80 to -0.05, p = .025). It is notable that three of the intervention components were significantly correlated with one another. Completion of the in-depth counseling session was associated with the number of pregnancy content calls received (r = .56, p < .0001) and the number of pregnancy podcasts downloaded (r = .51, p < .0001). The number of pregnancy content calls completed was also significantly associated with the number of pregnancy podcasts downloaded (r = .87, p < .0001). Visiting the Facebook group was not associated with the other pregnancy intervention components.

Six-month postpartum weight retention was not significantly associated with completing the in-depth postpartum counseling session (r = − .12, p = .28), number of postpartum calls completed (r = -.13, p = .24), number of postpartum podcasts downloaded (r = -.15, p = .17), or viewing or posting to the postpartum Facebook group (r = .03, p = .78). Completion of the in-depth postpartum counseling session was associated with the number of postpartum calls received (r = .80, p < .0001) and the number of postpartum podcasts downloaded (r = .42, p < .0001). The number of postpartum calls completed was also significantly associated with the number of postpartum podcasts downloaded (r = .53, p < .0001). Viewing or posting to the postpartum Facebook group was not associated with the other postpartum intervention components.

## Perceived helpfulness of program components – behavioral intervention group

The perceived helpfulness of each pregnancy and postpartum program component for the behavioral intervention participants is reported in Table 5.

**Table 5:** Perceived helpfulness of program components during pregnancy and postpartum, behavioral intervention participants

	Pregnancy (n=88)			Postpartum (n=78)		
Program Component	Possible Range	Mean (SD) or %		Possible Range	Mean (SD) or %	
**In-depth counseling session**						
Helpfulness of healthy eating information	^a^0-3	2.6 (0.6)		^b^1-3	2.4 (0.5)	
Helpfulness of exercise information	^a^0-3	2.4 (0.7)		^b^1-3	2.4 (0.6)	
**Telephone calls**						
Helpfulness of phone calls	^a^0-3	2.4 (0.8)			---	
Helpfulness of phone calls in						
supporting healthy eating				^b^1-3	2.4 (0.6)	
supporting exercise				^b^1-3	2.4 (0.7)	
supporting losing weight				^b^1-3	2.3 (0.7)	
Number of phone calls						
Too few		1.1			7.9	
Just about right		72.4			71.1	
Too many		26.4			21.1	
Call duration						
Too short		1.2			1.3	
Just about right		75.0			90.9	
Too long		23.8			7.8	
**Podcasts**						
Overall quality of podcasts	^c^0-4	2.9 (1.0)		^c^0-4	2.6 (0.9)	
Helpfulness of podcasts	^a^0-3	2.0 (0.8)		^b^1-3	1.9 (0.6)	
^d^Number of podcasts						
Too few		3.5			0	
Just about right		70.9			37.8	
Too many		25.6			62.2	
Accessing information in podcasts was easy	^e^1-7	6.7 (0.7)		^e^1-7	6.4 (1.0)	
Podcasts effective way to get information about exercise	^e^1-7	5.5 (1.6)		^e^1-7	5.0 (1.8)	
Podcasts effective way to get information about healthy eating	^e^1-7	5.8 (1.6)		^e^1-7	5.1 (1.8)	
Podcasts effective way to get information about weight control / weight loss	^e^1-7	5.5 (1.7)		^e^1-7	5.0 (1.8)	
**Facebook group**						
^f^Helpfulness of Facebook group	^a^0-3	1.6 (0.7)		^b^1-3	2.0 (0.6)	
^f^Helpfulness of staff posts to Facebook group	^a^0-3	2.0 (0.6)		^b^1-3	2.1 (0.5)	

^a^0 = did not help at all, 1 = did not help very much, 2 = helped some, 3 = helped a lot

^b^1 = did not help, 2 = helped some, 3 = helped a lot

^c^0 = poor, 1 = fair, 2 = good, 3 = very good, 4 = excellent

^d^10 podcasts in pregnancy and 16 podcasts in postpartum

^e^1 = totally disagree, 7 = completely agree

^f^Limited to participants who reported visiting Facebook group (n = 60 at pregnancy visit, n = 35 at postpartum visit)

### In-depth counseling session

Behavioral intervention participants rated the healthy eating and exercise content of both the pregnancy and postpartum in-depth counseling sessions as midway between “helped some” and “helped a lot” (2.4 to 2.6 out of 3).

### Telephone counseling

The telephone counseling calls in pregnancy and postpartum were rated similarly by behavioral intervention participants for their overall helpfulness (assessed in pregnancy) and for their helpfulness in supporting healthy eating, exercise, and losing weight (assessed in postpartum); midway between “helped some” and “helped a lot” (2.3 to 2.4 out of 3). Almost three-quarters of participants described the number of phone calls in pregnancy and postpartum as “just about right,” and while a similar number rated the pregnancy call duration (for calls 1 to 10) as “just about right” (75%), more rated the call duration as “just about right” for the shorter pregnancy calls delivered after the first 10 content-based calls (87%, data not shown) and for postpartum calls (91%).

### Podcasts

Behavioral intervention participants, on average, rated the overall quality of the podcasts as “very good” during pregnancy (2.9 out of 4) and between “good” and “very good” during postpartum (2.6 out of 4). They rated the helpfulness of the pregnancy and postpartum podcasts lower than the in-depth counseling sessions and the telephone calls, reporting that on average the podcasts “helped some” (2.0 and 1.9 out of 3). While most (71%) thought that the number of podcasts in pregnancy (10 total) was “just about right,” a smaller percentage described them in this way in postpartum (16 total) (38%), with more saying that there were “too many” (62%). Participants had high agreement that accessing information in the podcasts was easy (6.4 to 6.7 out of 7), and had moderate to high agreement that podcasts were an effective way to get information about exercise, healthy eating, and weight, with ratings somewhat more favorable in pregnancy (5.5 to 5.8 out of 7) as compared to postpartum (5.0 to 5.1 out of 7).

### Facebook

The Facebook groups were rated least favorably. On average participants described the helpfulness of the Facebook pregnancy group as between “did not help very much” to “helped some” (1.6 out of 3), and the helpfulness of the Facebook postpartum group as “helped some” (2.0 out of 3). Participants rated the staff posts to the Facebook group, on average, as “helped some” for both pregnancy (2.0 out of 3) and postpartum (2.1 out of 3).

## Perceived helpfulness of behavioral strategies - behavioral intervention group

The behavioral intervention emphasized the use of evidence-based behavioral strategies and activities, most consistent with Social Cognitive Theory. As shown in Table 6, the strategies that behavioral intervention participants reported using most often (used “most of the time” or greater; ≥ 2 out of 3) in pregnancy were setting healthy eating goals, setting exercise goals, weighing yourself, and wearing a pedometer. The strategies used least often (“sometimes” or less; ≤ 1 out of 3) were using the recipes shared by the study and asking people for support and help with eating healthy and with exercising. All other strategies were used more than “sometimes” but less than “most of the time.” The internal consistency of these items was high (Cronbach’s alpha = 0.81).

**Table 6:** Perceived frequency of use and helpfulness of behavioral strategies, behavioral intervention participants

	Pregnancy (n = 88)			Postpartum (n = 78)	
Behavioral Strategy	Possible Range	^a^Frequency of use, Mean (SD)	^b^Perceived helpfulness, Mean (SD)	Possible Range	^c^Perceived helpfulness, Mean (SD)
Using a log to keep track of eating	0–3	1.5 (0.9)	1.9 (1.0)	1–3	1.8 (0.8)
Using a log to keep track of exercise	0–3	1.6 (1.0)	1.9 (1.0)	1–3	1.9 (0.8)
Wearing a pedometer	0–3	2.0 (0.9)	2.4 (0.9)	1–3	2.3 (0.8)
Setting healthy eating goals	0–3	2.5 (0.7)	2.5 (0.6)	1–3	2.4 (0.6)
Setting exercise goals	0–3	2.3 (0.9)	2.2 (0.8)	1–3	2.2 (0.6)
Using a menu or shopping list to plan meals	0–3	1.7 (1.0)	2.2 (0.9)	1–3	2.3 (0.8)
Using problem solving strategies	0–3	1.5 (0.8)	2.1 (0.8)	1–3	2.1 (0.7)
Using stress management strategies	0–3	1.4 (0.8)	2.1 (0.8)	1–3	2.2 (0.7)
Using time management strategies	0–3	1.4 (0.8)	1.9 (0.9)	1–3	2.1 (0.7)
Reading food labels	0–3	1.8 (0.9)	2.2 (0.9)	1–3	2.3 (0.7)
Weighing yourself	0–3	2.2 (0.8)	2.4 (0.8)	1–3	2.5 (0.6)
Using recipes shared by HIPP	0–3	0.7 (0.6)	1.4 (1.0)	1–3	1.9 (0.7)
Asking people for support and help with eating healthy	0–3	1.0 (0.8)	1.5 (1.0)	1–3	1.9 (0.7)
Asking people for support and help with exercising	0–3	1.1 (0.8)	1.5 (1.0)	1–3	2.0 (0.7)
Using money saving strategies for buying food	0–3	1.5 (0.9)	1.9 (1.0)	1–3	1.9 (0.8)
^d^Mean score across strategies	0–3	1.6 (0.4)	2.0 (0.6)	1–3	2.1 (0.4)

^a^0 = never, 1 = sometimes, 2 = most of the time, 3 = always

^b^0 = did not help at all, 1 = did not help very much, 2 = helped some, 3 = helped a lot

^c^1 = none or did not do, 2 = some, 3 = a lot

^d^Cronbach’s Coefficient Alpha = 0.81 for frequency of use items; 0.86 for pregnancy helpfulness items; 0.89 for postpartum helpfulness items

Participants reported that most of the strategies in pregnancy “helped some” to “helped a lot” (≥ 2 out of 3). Strategies rated, on average, as lower than “helped some” (< 2 out of 3) included using recipes shared by the study and asking people for support and help with eating healthy and with exercising. Internal consistency of these items was high (Cronbach’s alpha = 0.86).

In postpartum, the questions about perceived helpfulness of strategies considered the frequency of their use in the response options, with the lowest rating of helpfulness being “none or did not do” (score of 1 out of 3). Ratings of perceived helpfulness were similar in postpartum as in pregnancy, with most strategies rated as helping “some” (≥ 2 out of 3). Using recipes shared by the study and asking people for support and help with eating healthy and with exercising were rated as somewhat more helpful in postpartum than in pregnancy. Internal consistency of these items was high (Cronbach’s alpha = 0.89).

## Standard care group

### Mailings

All mailings were pre-scheduled and sent to participants accordingly. Across mailings, standard care participants on average reported that they read “a lot” of the materials (0 = did not read, 1 = read a little, 2 = read a lot, 3 = read it all), with mean scores of 1.9 and 2.1 out of 3 during pregnancy and postpartum, respectively. Across mailings, participants on average rated the materials as “somewhat helpful” to “very helpful,” (0 = not at all helpful, 1 = not very helpful, 2 = somewhat helpful, 3 = very helpful) with mean scores of 2.3 and 2.4 out of 3 during pregnancy and postpartum, respectively. Internal consistency of the items for their report of reading mailings and the perceived helpfulness items was high in pregnancy (Cronbach’s alpha = 0.95 and 0.96) and postpartum (Cronbach’s alpha = 0.97 and 0.97).

### Podcasts

Standard care participants, on average, rated the overall quality of the podcasts as “very good” during pregnancy and between “very good” and “excellent” during postpartum, with mean scores of 3.1 and 3.3 out of 4, respectively. Across podcasts, participants rated the podcasts as between “somewhat helpful” and “very helpful,” with mean scores of 2.3 during pregnancy and 2.4 out of 3 during postpartum. While most participants (75%) thought the number of podcasts in pregnancy was “just about right,” fewer described them in this way in postpartum (66%), with more saying that there were “too many” (26%). Participants had high agreement that accessing information in the podcasts was easy during pregnancy and postpartum, with mean scores of 6.5 and 6.2 out of 7, respectively, and had moderate to high agreement that podcasts were an effective way to get information about pregnancy (mean score of 6.1 out of 7), and about caring for one’s baby and learning about parenting (mean score of 5.9 out of 7).

## Open-ended responses – behavioral intervention group

To highlight the behavioral intervention participants’ most salient and most frequent likes and dislikes, this section describes codes that were used 9 or more times in pregnancy and 8 or more times in postpartum (i.e., > 10% of participants). Within each of the following sections, the most frequently coded theme is listed first and ordered by frequency. These analyses are limited to the behavioral intervention participants only.

### In-depth counseling session

Behavioral intervention participants most frequently described the nutrition information as the most helpful aspect of the in-depth counseling session. For example, “It really broke down what I was eating and I was able to make adjustments” and “learning about exactly how much dairy, protein, veggies, fruits, etc. you actually need during pregnancy.” Other themes coded 9 or more times as most helpful included the accountability that HIPP provided (“accountability, suggestions to fit personal life, handouts”), goal setting (“having someone help me set goals”), general information (e.g., “the information given”), support provided (“one-on-one help – very personalized”), and positive characteristics of the staff (“my counselor explained everything very well and helped me set goals”). By far, the most common response to what participants found least helpful was “nothing,” cited by 36 participants. No other themes were coded 9 or more times in response to what was least helpful.

Themes for the in-depth postpartum counseling session were similar, with support, nutrition information, general information and advice, and accountability coded 8 or more times as most helpful, and “nothing” as least helpful. Participants also cited phone calls as helpful, indicating that perhaps participants did not differentiate between the in-depth counseling session and phone calls in response to this question.

### Telephone calls

Behavioral intervention participants most liked the support that telephone calls during pregnancy provided, for example, “(Name of Counselor) encouraged me to keep up the good work and not to give up. She was very positive.” They also described the accountability that regular calls provided, “I liked that it kept me motivated to complete my goals and held me accountable.” Reference to general information or advice (“They’re informative”), setting goals, and the staff were also frequently cited as most liked. Like the counseling session, the most common response to what participants liked least about telephone calls was “nothing,” noted by 30 participants. Two aspects of telephone calls during pregnancy that participants least liked were that the call duration was too long (“the calls could be less time”) and the information was repetitive, particularly with other components of the intervention (“Sometimes repetitive with podcasts, handouts, and call”).

Similar themes were seen for the postpartum telephone calls. Participants most liked the accountability that the calls provided, the support they received, characteristics of the staff (“very knowledgeable and comforting”), and goal setting. In response to what they liked least about the postpartum telephone calls, “nothing” was the most common response, cited by 30 participants. A smaller number of participants reported issues related to scheduling the calls as least liked, for example, “trying to find time to talk” and “scheduling them.” No other least liked responses were coded 8 or more times.

### Podcasts

Behavioral intervention participants cited that they most liked the general information or advice provided in the pregnancy podcasts, for example, “I enjoyed the information provided in the podcasts.” Participants also described the podcasts as relatable (“Real situations help to relate to what I was going through (for food, work, etc.)”), they liked the storylines (“I looked forward to hearing about what other mothers experienced, challenges, solutions, and outcomes”), and they saw them as realistic (“Real people, real experience”). The most common response to what participants liked least about the pregnancy podcasts was “nothing,” noted by 25 participants. Only one aspect of the podcasts was cited by 9 or more participants as least liked: the podcast duration was too long. Sometimes this theme was cited in combination with being repetitive, “kind of long and most of the information was in the handouts too.”

Participants most liked the general information and advice and how relatable the postpartum podcasts were (“Hearing other moms’ experiences and what worked well for them”). In terms of what was least liked, the most common response was “nothing,” cited by 20 participants, followed by the duration being too long and having too many postpartum podcasts.

### Facebook

Behavioral intervention participants most liked the general information and advice provided on the pregnancy Facebook group (“There were good articles posted”), the recipes (“Loved recipes and exercise videos/ideas; will keep long after the study is over”), and the exercise workouts (“The yoga and exercise posts were my favorite”). By far, the least-liked aspect of the Facebook group was the lack of interaction between participants, for example, “Not many women interreacted with one another.” Within this context, some noted that they were reluctant to post on Facebook because other participants were not doing so, “Seemed like no one was responding to discussion posts. I didn’t want to be the only one to respond.” No other aspects of the Facebook group were cited by 9 or more participants as least liked.

Based on the feedback provided during the 32-week process evaluation survey, at postpartum we asked participants to share their thoughts or suggestions for the Facebook group and to share their reasons for not participating in the postpartum Facebook group. The most common suggestion was to increase interaction between participants, but few concrete ideas were provided. Examples of common responses were “There weren’t enough members interacting to be of much help” and “I feel like you need more people to give it a more community feeling.” The most common reasons provided for not participating were that they are not active on Facebook and there was not adequate interaction.

### Other

In response to whether the participant wished to share anything else about the study, the most common response during pregnancy was that they had a good experience with the program. Examples of these comments included, “Great program!” and “I enjoyed my time & hoping to continue using the new knowledge that I’ve learned. I think this study was great to holding me accountable & a step in the right direction for future success in my health.”

That same theme was seen in the postpartum responses, for example, “Thank you for the opportunity. I learned so much going through this my 2nd pregnancy. I would’ve loved to have this the first time. The next child will definitely benefit from my knowledge this time!” When describing their positive experience, they commonly cited staff as contributing to this experience, “Great job! Staff was extremely helpful. Thank (name), (name), and (name).”

### What would have facilitated greater participation?

The most common response to question of what would have helped the participant take part in the program more fully was “nothing,” cited by 17 participants. Some women discussed barriers rather than enablers – for example, one theme was that competing priorities made participation difficult. For example, “It was a hard transition for me to go from being a mom of 1 to a mom of 2. We don’t have any family and not many friends to help out so it makes it harder for me to focus on anything other than my kids and husband.” Or “For me it’s time, I am in the middle of a relocation, working full-time and caring for 3 small children.” Some women recommended having group meetings as part of the program, either as a formal group (“meeting in person getting to know other participants, making it personal, and real instead of business like”) or more informal meet-ups (“Maybe pair up individuals? Just to increase support and camaraderie”). Two other themes were coded 7 times each and are thus reported to err on the side of inclusion. They were that the lack of time hindered participation, which was like the theme of competing priorities, and the suggestion to increase accountability, for example by requiring that tracking logs be turned in.

## Discussion

The HIPP study was unique in that it focused on women who entered pregnancy living with overweight and with obesity, included a large proportion of African American women (44%), was theory-based, followed women from pregnancy (mean of 12.6 weeks gestation) through postpartum, and incorporated novel intervention components (Facebook, podcasts). It is also unique in its conduct of a comprehensive process evaluation. This in-depth process evaluation revealed important learnings that could inform future interventions. Overall, both intervention and standard care participants rated the HIPP study components favorably, and over 90% of participants in each group would recommend the study to a friend. Participants’ positive experiences with the study likely led to the acceptable completion rates of the 32-week pregnancy and 6-month postpartum process evaluation surveys.

There are several limitations that should be considered when interpreting our study findings. First, we recruited a well-educated sample of women; results may not generalize to less educated women. Second, participants who were less engaged in the intervention were also less likely to complete the process evaluations surveys. Thus, our findings may be biased toward those who had more favorable study experiences. However, many of these participants with very low intervention engagement were lost early in the study, likely before they had much exposure to the intervention. Third, we did not objectively capture Facebook engagement, and thus were unable to report factors such as views, likes, and posts. Fourth, although we were able to objectively capture if podcasts were downloaded, we were unable to measure listening time. Finally, a higher percentage of standard care than behavioral intervention participants completed the pregnancy and postpartum process evaluation surveys, perhaps due to the overall time and burden of the behavioral intervention components (described later).

This study provided useful data regarding which intervention components were preferred by pregnant and postpartum women and some reasons for these preferences. Overall, participants rated the two in-depth counseling sessions (pregnancy and early postpartum) and the telephone counseling calls as somewhat more helpful than the podcasts and the private Facebook groups. Qualitative data from our study suggest that these components, which were delivered directly by staff to the participant, were valued in large part because they created accountability, were viewed as supportive, and facilitated goal setting. About three-quarters of participants found the number of calls and the call duration to be “just about right,” with most of the remainder preferring fewer and shorter calls. Furthermore, both completion of the in-depth pregnancy counseling session and the number of the content-based calls delivered in pregnancy related to more favorable (i.e., less) gestational weight gain, providing further evidence for the effectiveness of these intervention modes. The use of telephone-based interventions to produce physical activity and dietary change is well-established in general populations [29, 30], and despite the younger age of our population, it was an intervention channel that appeared to be well-received. It is worth noting, however, that the average age of participants who received 9 or 10 of the 10 content calls were about 2.5 years older than women who received fewer than 9 calls.

The podcast and Facebook components of the intervention were used to provide a convenient, efficient, and contemporary way for participants to receive support for their lifestyle changes. Many pregnant and postpartum women are busy with family and occupational responsibilities, and we wanted to give women who had difficulty engaging in telephone calls a way to still access intervention content. The podcasts reinforced information covered on telephone counseling calls and were modeled after an earlier study that showed meaningful weight loss with a podcast-only approach [25]. HIPP participants rated the podcasts very high on their ease of access and moderately high in their effectiveness and quality, but only moderate in their helpfulness. There are several reasons why participants might have rated the podcasts as less helpful than the phone calls. Responses to open-ended questions indicated that participants did not like the redundancy in content between calls, handouts, and podcasts. In fact, the number of calls received was highly correlated with the number of podcasts downloaded, and both were significantly related to more favorable (i.e., less) gestational weight gain, indicating that more engaged participants were receiving both intervention components. Because the counseling calls were scheduled, involved human contact, did not require initiative from the participant (i.e., staff called the participant), and had content that overlapped with the podcasts, participants might have been less motivated to download and listen to the pregnancy podcasts. During postpartum, when women were busy caring for their new baby, podcast downloads were even lower. Future studies might wish to compare telephone only versus podcast only for effectiveness. An adaptive design, such as the stepped care model being tested by Symons Downs et al. [31], could be employed that begins with podcasts (less staff burden) and introduces telephone calls only if participants are not meeting study goals. Alternatively, a hybrid approach with low personnel burden could be tested that combines podcasts with an automated phone intervention (e.g., interactive voice response system) that allows for scheduled calls, interactivity, accountability, and the use of phone contacts in a cost-effective manner [32–34]. Finally, postpartum podcasts were more likely to be downloaded by those of higher socioeconomic status, participants who were somewhat older, and by those who were married (and thus perhaps had more financial resources and/or support). Various factors could explain lower utilization in lower socioeconomic status participants including lack of time.

The original intent of the Facebook groups was to provide informational resources, empower women to choose healthy behaviors, and encourage social support between intervention participants. Facebook was chosen as the platform to facilitate group social support because it is the most popular social media platform among women 18–40 years old [35]. In addition, our prior work showed that in interventions among non-pregnant adults [36] and women experiencing fertility and trying to become pregnant [37], engagement with Facebook groups during a behavioral intervention was associated with weight loss. In the HIPP trial, weekly study posts to the Facebook group included recipes, workout videos, and Q&A prompts to engage participants. When it was clear that engagement (likes and posts) was low, we attempted to facilitate more interaction by creating active participation posts such as using the polling function and asking women to share a photo (e.g., nursery or “bump” growth update), features that yielded more engagement in our prior work [36]. These attempts, however, had little impact, and participants cited the lack of interaction in the groups as the least liked aspect of this intervention component.

Prior research has shown intervention components can be delivered via Facebook for postpartum women with active and sustained engagement in about half of the total participants enrolled [38–40]. Similar to our findings, Baruth et al. [41] found that in their multicomponent intervention for pregnant women, the Facebook group was not frequently used and was not perceived to be very helpful. It is possible that our rolling recruitment interfered with forming a sense of cohesion as women were added and removed from the group at different times instead of as cohorts. Further, participants did not have opportunities to get to know each other prior to being in a Facebook group together. Occasional in-person events might have fostered more or a sense of community. Given they did not have existing relationships, it is possible that if participants had been completely anonymous in the Facebook group, as is common in parenting forums [42], they might have felt more comfortable making self-disclosures and engaging with posts. Furthermore, participation in the Facebook group was optional and not incentivized, and in response to open-ended questions, some participants cited that they did not use Facebook in general. The HIPP Facebook groups were used more often by higher income, more educated, and married participants. The combination of these factors might have made the number of active participants in a group at one time too low to ensure active participation. Finally, the lack of social engagement on Facebook does not mean that participants received no benefit from this intervention channel. “Lurkers,” or those who do not actively participate on social media by posts/likes, have been shown to benefit as much from online support groups as those who post [43]. Indeed, participants rated the helpfulness of staff posts to Facebook group as more favorable than the group itself, and in response to open-ended questions, cited the general information, recipes, and exercise videos as most helpful. Nonetheless, visiting the Facebook group was not associated with study outcomes.

Our pilot formative work indicated that women wanted to come together with other women in a group setting to learn how to be more active and eat healthier during pregnancy and postpartum [23]. Yet, results from our earlier pilot study and our experiences at the beginning of the HIPP trial indicated that attendance at group classes was low, likely due to the participants’ busy schedules, and the realities of recruiting an adequate number of pregnant women at one time made this design infeasible. Nevertheless, in the open-ended responses, participants expressed an interest in coming together with other participants. Future studies might wish to hold live sessions designed to foster support and cohesion, even if held virtually (e.g., videoconferencing) or in-person occasionally. Experiences with COVID-19 might make remote delivery of content via platforms such as Zoom more feasible and acceptable. Live contacts (virtual or in-person) might also foster greater Facebook engagement by making participants feel more connected to the study and to other participants.

Lastly, it was more difficult to engage participants in the postpartum period as compared to the pregnancy period. The dose received of each intervention component was higher in pregnancy than in postpartum: in-depth counseling session (94% vs. 78%), call completion (79% vs. 53–61%), podcast downloads (72% vs. 33%), and visiting/viewing the Facebook group (68% vs. 45%). This finding is perhaps not surprising, as postpartum women are adjusting to life as a mother (or adding to their existing family) and the demands that a newborn places on them, while often having to resume work and other responsibilities. Intervention approaches that can more effectively engage postpartum women for healthy lifestyle changes are needed.

## Lessons learned

This study showed that telephone counseling is a desirable lifestyle intervention channel for pregnant and postpartum women due to its perceived helpfulness and favorable association with gestational weight gain. It created accountability, lent support, and fostered goal setting. Private Facebook groups, as used in the HIPP study, do not seem particularly helpful or liked and, if used in future programs, may require additional efforts to increase cohesion and connection through in-person or virtual live events. Podcasts, a novel approach tested in this study, related favorably to gestational weight gain, but were perceived as redundant with phone content. Although providing intervention content in multiple formats has the potential to accommodate a variety of different learning styles, it is possible that providing redundant information via numerous different channels led to an increase in extraneous cognitive load [44]. Future studies may wish to limit the intervention components or use an adaptive, stepped care approach to add components as necessary.

## Data Availability

The datasets generated and/or analyzed during the current study are not publicly available due but are available from the corresponding author on reasonable request.
